# Medical Opinion and Movement

**Published:** 1908-05-23

**Authors:** 


### Medical Opinion and Movement.

THE proper surgical treatment of abscesses, fistulous tracts, and sinuses depends to a large extent upon an accurate knowledge of the extent of the disease. Even when a thorough exploratory operation is undertaken, it is possible to miss some of the more hidden ramifications of an abscess tract. In order to determine the exact anatomical topography of such a condition Dr. E. G. Beck of Chicago recommends the injection of a bismuth-vaseline paste into the cavity, followed by an examination of the part with the *x*-rays. Owing to the impermeability of the bismuth to the rays a radiograph is obtained of the cavity and its ramifications. The paste is made up of 30 parts of bismuth subnitrate and 60 parts of vaseline. But Dr. Beck also uses this paste for remedial treatment as well as for diagnostic purposes. He finds that the injection of this paste has a most beneficial effect upon these chronic abscess cavities and sinuses, especially of a tuberculous nature. He has tried the method in fourteen cases, and all have been cured within a short period. Some of them were extremely chronic, and had discharged pus for a year or more. The cavities must be thoroughly dried first by packing with gauze. The paste is sterilised and is forced into the sinuses while still hot with a glass syringe. When the discharge ceases Dr. Beck uses a harder mixture containing in addition 5 parts of white wax and 5 parts of soft paraffin. In some cases 1 per cent. formalin may be added.

DR. L. BOLTON BANGS of New York gives an account of three cases in his experience which led him to believe that in many instances restlessness, irritability, melancholia, and even symptoms simulating collapse following operations, may be due to the sudden withdrawal of tobacco from patients who have acquired the tobacco habit in a marked degree. Two of the patients reported by the author were strong *habitués*, while the third was only what would be called a moderate smoker. In all three instances the symptoms were such as to cause alarm, so that morphia and strong stimulants were resorted to before the condition was recognised. The smoking of a cigarette was in each instance, however, followed by the prompt cessation of all symptoms. Tobacco is almost invariably interdicted in all cases of serious illness whether calling for ordinary medical or surgical intervention, and the patient usually shows no desire for the "weed" until convalescence is well advanced. On the other hand, tobacco is undoubtedly of the nature of a narcotic drug, and it is well known that its sudden withdrawal from a habitual smoker causes considerable discomfort and physical and mental disturbances. It would be advisable to bear these experiences in mind, and not be too dogmatic in the denial of tobacco to patients who are habitual smokers, and who have no symptoms which definitely contraindicate the indulgence.

SCHELBE, in the *Münich Med. Wochensch*, describes a method of approximately estimating the absorption of fat in infants, which, although only practicable in hospital practice, is yet of sufficient interest to be mentioned in view of the importance of gastro-intestinal pathology in children. A few cubic centimetres of blood taken from the infant under observation are centrifugalised, defibrinated and examined for emulsified fat, which is shown to be present by the turbidity of the serum. The intensity of the turbidity is directly proportional to the fat dissolved. Serum obtained from fasting infants is always clear, and contains no fat. There is a similar absence of fat when the infant is fed on hydrocarbons or on milk from which all cream has been separated. When the diet consists of human or cow's milk the serum is turbid during the second and third hour following the meal, the maximum absorption of fat taking place during these hours. Children suffering from gastro-intestinal affections, especially diarrhœa and vomiting, show no turbidity in their serum, even though their diet be particularly rich in fats, and, moreover, the "ring" test with osmic acid is negative when applied to the serum. The method is, of course, only approximately accurate, since no account is taken of the fat which passes to the liver *via* the portal vein, nor of such fat as is dissolved in the serum of the tissues. Schelbe's researches will not close the discussion as to the prognostic value of insufficient absorption of fat in the gastro-enteritis of children, but they are a step towards the solution of the problem.

THE treatment of chorea by drugs, so often discussed, was dealt with by Dr. Leonard Guthrie in a recent paper before the Therapeutical Section of the Royal Society of Medicine. He shares the belief of many physicians in the all-importance of regimen and diet in this disease, and in the uselessness of drugs except as palliatives. He does not appear to favour the administration of equal or double doses of sodium bicarbonate together with salicylate of soda, a measure which has been praised by many competent observers, among them Dr. Lees, who advocates the use of very large doses of salicylate given in this way. To give poison and antidote together seems to Dr. Guthrie to be a questionable therapeutic measure. Arsenic also, prescribed according to the "intensive" method, was not recommended in the paper, although in the discussion which followed it was evident that this treatment of chorea is exceedingly popular in Scotland and the North of England, and a suggestion was made that environment has some influence upon the reaction of choreic patients to arsenic. Dr. Cecil Wall spoke very strongly in favour of the administration of aceto-salicylic acid in chorea. According to his statistics 92 per cent. of cases treated thus were discharged from hospital cured in less than two months, while the majority of these were cured in four weeks from admission. Two months' treatment by arsenic or salicylate of soda produced a cure in only 38 per cent. To avoid the possible dangers of vomiting aspirin must not be given on an empty stomach, and it should only be given in powder form. For violent and severe cases of chorea the hypnotics suggested by Dr. Guthrie are chloral and bromide, chlorolamide, or chloretone. Dr. Wall recommends chloral or chloralamide, and deprecates the use of bromides, which he has known to produce mania. It is the experience of some physicians that chloretone is a remedy which may lead to profound depression, without otherwise influencing the course of the disease. The general opinion at the present day, at least in London, seems to be that of drugs, salicylates, in some form or other, are best suited for routine administration in chorea. This is in accordance with the modern view that chorea is a true rheumatic manifestation.

---

No one would depreciate the many and rapid advances that have been made in the sphere of bacteriology and their importance in the treatment of disease; but it must be admitted that the general practitioner is compelled for many reasons to view the progress at a distance, without forthwith assimilating and utilising all the means and methods which it offers in his daily routine. The brilliant work of Sir A. E. Wright on the treatment with vaccines controlled by the opsonic index involves too much labour of a highly technical nature in its application to allow of general adoption in the practice of medicine. There is, moreover, the personal factor in regard to the patient to consider. A prick of the finger for blood examination or a hypodermic injection are sufficiently simple procedures, but are apt to be resented, especially when repeated too frequently, and one can well imagine the unenviable reputation which might be created by an over-zealous doctor, who in the course of his round pricked one patient's finger for a blood count, a second's for the opsonic index, injected serum into a third, performed lumbar puncture on a fourth, and carried out Calmette's ophthalmo reaction on a fifth! It is interesting, therefore, to find that there is a very definite endeavour to simplify some of these latest methods and bring them more within the limits of practical politics. Dr. Arthur Latham, in a recent communication to the Royal Society of Medicine, brings forth evidence to show that tuberculin may be given by the mouth with satisfactory results, and that the effect of the treatment can be gauged by the variations in the temperature and by the condition of the patient, without resort to opsonic estimations. A rise of temperature corresponds to a fall in the opsonic index, and indicates too large a dose, and this is also accompanied by symptoms of malaise, loss of appetite, and so forth. Such is briefly the satisfactory result of his investigations. For further details as to dosage and methods of administration we must refer our readers to the paper, which is well worthy of perusal.

---

Lumbar puncture is yet another means of diagnosis and treatment, which has in recent years been placed at the disposal of the profession, but which the general practitioner does not put into practice with a light heart. As a means of treatment it is undoubtedly called for in certain conditions, and should not be avoided; but as a method of diagnosis only one may rightly hesitate to employ it. In epidemic cerebro-spinal meningitis it has been extensively employed for both diagnostic and therapeutic purposes, but it is satisfactory to know that we now have other methods at our command for diagnostic purposes. We refer to the meningococcal clumping reaction and to the opsonic index. For these estimations only a little blood-serum of the patient is necessary. When the blood-serum of a patient suffering from the disease is mixed with an emulsion of a meningococcus culture on agar a clumping reaction is obtained similar to that obtained with the specific cocci in Malta fever. Normal serum shows a very low opsonic index to the meningococcus. But in cases of cerebro-spinal fever it is by comparison remarkably high. The opsonic index is, acording to Dr. Houston and Dr. Rankin, more delicate and certain than the clumping reaction, and the two taken together constitute a specific test for the meningococcus which is of great value in diagnosis. It fails only in those rare cases which may be termed fulminating; but after the sixth day it is claimed to be of even greater value in diagnosis than lumbar puncture.

---

It is a belief of long standing that white races cannot flourish and breed successfully in the tropics. Even in those parts of the West Indies and South America, where exceptions may be quoted, a good deal of fresh immigration, and of infusion of black blood, has been necessary in the process. It has, therefore, not unnaturally been concluded that there is some climatic influence in the tropics which is prejudicial to the health and fecundity of Europeans and their descendants. While tropical diseases were attributed to some mysterious taint in tropical air, there was little objection to that conclusion; but since the development of tropical science and hygiene the question arises anew whether there is really anything more than parasitic disease which militates against the propagation of white stocks in those regions. The answer must be to some extent sought statistically, and cannot be completely furnished for some generations yet, but the prevailing impressions among trained observers are, even at this early stage, of some value.

---

It was said a few years ago by Dr. Ahearne, of an essentially tropical region, North Queensland, that white children born and bred there grow weedy and lanky, and that degeneration and loss of vitality rapidly appear in succeeding generations. This dismal prospect is one which Dr. T. P. MacDonald thinks will now be averted. In a paper read before the Society of Tropical Medicine and Hygiene he compares the appearance and physique of the "native born" in the Geraldton district of North Queensland before and after the introduction of tropical hygienic and prophylactic measures. He is enthusiastic on the physical perfection, energy, and vitality of the young people there: sunlight and exercise were encouraged by a system of picnics, rides, races, boating parties, and so on, for both sexes, and it is now established to Dr. MacDonald's satisfaction that white races can do the necessary manual labour of farming in the rainiest and one of the hottest climates on the globe. Indeed, it is manual labour that he regards as one of the most important factors in tropical hygiene for white men and women. The author did not touch on the decreased fecundity of whites so often described in the tropics. Whether this, if it really exists, is due, as some believe, to the deleterious effect of prolonged courses of quinine on the cells of the reproductive organs is a point on which the progress of mosquito eradication ought presently to throw some light. It is a great thing to render the tropics habitable for Europeans in comparative immunity from disease, but if it shall be proved that they need also suffer no loss of fertility there, a very much greater field is thrown open to permanent colonisation.

---

With a view to testing the efficiency of atropine as a restorative in chloroform poisoning, or, as a precautionary measure before anæsthesia, W. Webster, of the University of Manitoba, has lately undertaken a series of experiments which tend to show that the accepted views upon the physiological action of atropine are in certain important particulars incorrect. Deductions drawn from experiments upon animals, under the more or less artificial conditions of laboratory anæsthesia, are of course, open to objections from the standpoint of the clinician. But the suggestion of administering atropine as a prophylactic and as a curative measure against chloroform poisoning emanated in the first instance from the laboratory; and therefore it is only right to treat with respect any subsequent criticism of this method which proceeds from the same source. Webster's experiments, the results of which are communicated to the *Bio-chemical Journal*, were made for the most part upon dogs. Contrary to the accepted teaching, they showed that the injection of atropine into the circulation never produced a rise of blood-pressure, but whenever any effect was produced it was in the direction of a fall of blood-pressure, preceded sometimes by a slight preliminary rise of short duration. Again, the chief and lasting effect upon the heart was found to be a diminution in the extent of movement, and not an augmentation of the heart-beat. The gradual establishment of tolerance to atropine has often been demonstrated; but Webster has been able to produce rapid tolerance within the limits of time of a single experiment lasting about an hour and a half. In this respect atropine, hyoscine, hyoscyamine, duboisine, daturine, and scopolamine behave alike. Further, the serum of an animal which has been rendered immune to large doses of these drugs will, if injected into another animal, confer a similar immunity upon it.

---

The practical application of Webster's physiological researches to the therapeutics of chloroform poisoning lead him to the conclusion that atropine has no beneficial effect upon this condition. He believes that adrenalin would produce more benefit; but, since the effect of this substance is transitory, frequent small doses should, as a rule, be administered. If the heart has not completely stopped, he says, adrenalin causes more active contractions, and consequently a rise of blood-pressure, which a few doses at short intervals may make permanent. This effect is produced by stimulation of vasomotor nerve-endings in various parts of the body. The restoration of heart-beat by intravenous injection of adrenalin could not be obtained after the heart had ceased absolutely; but could be obtained when the heart movements, although no longer to be felt through the chest wall, could just be observed when the thorax was opened. Webster concludes that the drugs of the atropine series, whilst they certainly eliminate the tonic cardio-inhibitory action of the vagus, have also a paralytic effect upon the heart, diminishing the output, which renders them almost useless in the treatment of chloroform poisoning. The injection of a solution of suprarenal extract, on the other hand, seems to him to be of practical utility, though to a limited extent, in this condition. Webster's conclusions are thus of the nature of destructive criticism, and tend, if anything, to show that the sudden appalling catastrophe of cardiac failure during chloroform anæsthesia is even more difficult to avert or to remedy than is usually taught in the text-books.

---

Additional aids to the diagnosis of pericardial effusion are always worthy of consideration. As long ago as 1878 Rotch showed that in the early stages of effusion the fluid collects in the lower part of the pericardial sac, especially at the right edge of the sternum, and that dulness here in the fifth interspace is often the first sign of pericardial effusion. Fourteen years later Ebstein corroborated these statements, and designated the angle formed by the right border of heart dulness and the upper border of liver dulness the "cardiohepatic angle." Under normal conditions this is a right angle; but in pericarditis it is obtuse, owing to displacement of the lung to the right. W. J. Calvert discusses this sign in the present number of the "Johns Hopkins Hospital Bulletin." Enlargements of the heart itself, except when due to mitral stenosis, rarely displace the anterior border of the right lung sufficiently to give an appreciable area of absolute dulness at the right border of the sternum; and enlargement of the heart, even from mitral stenosis, seldom causes appreciable dulness in the fifth right interspace. The author states that in pericarditis three factors operate to produce dislocation of the lung at this point: (1) Possible displacement of the root of the lung; (2) pressure of the distended pericardium on the lung from its root to its lower border; and (3) downward displacement of the attachment of the right wall of the pericardium to the diaphragm, thereby lowering the point of greatest displacement of the right wall of the pericardium. In enlargement of the heart these factors are usually absent. In enlarged hearts the lesion must be determined before an increased cardio-hepatic angle can be regarded as clinical evidence of an associated pericardial effusion. In extreme dilatation of the heart, when differentiation from pericarditis is the most difficult, the cardio-hepatic angle is of little value, unless the history of the case is known; even then it is, at best, uncertain. When heart lesions can be excluded, increase in the angle is a valuable sign of pericarditis with effusion.

